# High Rab27A expression indicates favorable prognosis in CRC

**DOI:** 10.1186/s13000-015-0303-3

**Published:** 2015-06-13

**Authors:** Chuanbing Shi, Xiaojun Yang, Yijiang Ni, Ning Hou, Li Xu, Feng Zhan, Huijun Zhu, Lin Xiong, Pingsheng Chen

**Affiliations:** Department of Pathology and Pathophysiology, School of Medicine, Southeast University, Nanjing, 210009 China; Department of General Surgery, the Second Affiliated Hospital of Nanjing Medical University, Nanjing, 210011 China; Department of Traumatic Surgery, Changzhou No. 2 People’s Hospital Affiliated with Nanjing Medical University, Changzhou, 213000 China; Department of Pathology, Jiangsu Cancer Hospital, Nanjing, 210000 China; Department of Hepatobiliary and Laparoscopic Surgery, YiXing People’s Hospital, the Affiliated YiXing Hospital of Jiangsu University, Yixing, 214200 China; Department of Pathology and Laboratory Medicine, the Affiliated Hospital of Nantong University, Nantong, 226000 China; Department of Pathology, the Second Affiliated Hospital of Nanjing Medical University, Nanjing, 210011 China; The Key Laboratory of Cancer Biomarkers, Prevention & Treatment Cancer Center and The Key Laboratory of Antibody Technique of Ministry of Health, Nanjing Medical University, Nanjing, 210029 China

**Keywords:** Rab27A, CRC, qPCR, IHC, Metastasis, Prognosis

## Abstract

**Background:**

Rab27A is a peculiar member in Rab family and has been suggested to play essential roles in the development of human cancers. However, the association between Rab27A expression and clinicopathological characteristics of colorectal cancer (CRC) has not been elucidated yet.

**Methods:**

One-step quantitative real-time polymerase chain reaction (qPCR) test with 18 fresh-frozen CRC samples and immunohistochemistry (IHC) analysis in 112 CRC cases were executed to evaluate the relationship between Rab27A expression and the clinicopathological features of CRC. Cox regression and Kaplan-Meier survival analyses were performed to identify the prognostic factors for 112 CRC patients.

**Results:**

The results specified that the expression levels of Rab27A mRNA and protein were significantly higher in CRC tissues than that in matched non-cancerous tissues, in both qPCR test (*p* = 0.029) and IHC analysis (*p* = 0.020). The IHC data indicated that the Rab27A protein expression in CRC was statistically correlated with lymph node metastasis (*p* = 0.022) and TNM stage (*p* = 0.026). Cox multi-factor analysis and Kaplan-Meier method suggested Rab27A protein expression (*p* = 0.012) and tumor differentiation (*p* = 0.004) were significantly associated with the overall survival of CRC patients.

**Conclusion:**

The data indicated the differentiate expression of Rab27A in CRC tissues and matched non-cancerous tissues. Rab27A may be used as a valuable prognostic biomarker for CRC patients.

## Background

Colorectal cancer (CRC) is the third most common cancer and more than 1 million people develop CRC annually [1]. It is reported that there are estimated 140,000 newly diagnosed cases of CRC and approximately 50,000 cancer deaths from CRC in America in 2013 [2, 3]. Of patients with primary CRC, nearly 20 % encounter distant metastasis at the time of diagnosis and only 10-30 % of patients with distant metastasis can have potentially curative resection of the primary tumor and the distant focus [4–6]. For CRC prognosis, survival is critically related to stage at diagnosis, with five-year survival rates of 90 % for localized cases, while 67 % for regional cases and only 10 % for distant metastatic cases [7]. Although the great developments for diagnosis and treatment for CRC, the overall survival rate of CRC patients rarely shows the encouraging result and post-operative recurrence and metastasis remain the two most challenging obstacles [8]. Hence it is valuable and necessary to identify molecular predictive markers for the prognosis, which would facilitate the selection of therapeutic strategies and further ameliorate patients’ survival for CRC [9].

The Rab family is a group of Ras-like small GTPases which locates in specific subcellular organelle and plays an important role in cell secretion, endocytosis, signal transduction, and development [10–12]. Rab27A is a peculiar member in Rab family for its specific role in human hereditary disease and dysfunction, such as Griscelli syndrome [13, 14]. Recently, several studies reported the diverse function of Rab27A in many kinds of human cancers. Differential expression of Rab27A was detected in murine xenografts of breast cancer (BC) metastasis and Rab27A was highly associated with the invasive and metastatic potential of human BC cells [15]. Rab27A enhanced various ability of glioma cell, such as developing proliferation, promoting invasion and inhibiting apoptosis [16]. Rab27A also affected exosome secretion, modified tumor microenvironment and finally promoted tumor progression [17]. Moreover, two studies reported the prognostic role of Rab27A that high Rab27A expression indicated poor survival of patients with gliomas and hepatocellular carcinoma (HCC) [18, 19]. All the above information suggest the significant relationship between Rab27A and cancer while the performance of Rab27A in CRC has yet to be explored. What function does Rab27A play in CRC deveolopment and what is the relationship between Rab27A expression and clinical features of CRC? We conducted this present research.

In this retrospective study, the mRNA and protein expression of Rab27A in CRC was investigated by one-step quantitative real-time polymerase chain reaction (qPCR) test and immunohistochemistry (IHC) analysis respectively. Subsequently, the correlations of Rab27A expression with the clinicopathological parameters of CRC were further explored. Finally, the survival analysis for identifying prognostic factors was executed.

## Methods

### Ethics statement

The Ethics Committee of the Affiliated Hospital of Nantong University and each local hospital approved the study protocol. A written informed consent and related pictures were also acquired from each patient for publication of this present research.

### CRC patient specimens

Formalin-fixed, paraffin-embedded tumor samples from 112 CRC cases and 113 matched non-cancerous tissue specimens were collected in CRC patients from the Department of Pathology, the Affiliated Hospital of Nantong University from 2003 to 2008. Representative and important clinical information were collected from hospital medical records, including gender, age, tumor size, tumor location, histological type, tumor differentiation, serum carcino-embryonic antigen (CEA) level, primary tumor (T), lymph node metastasis (N), distant metastasis (M), as well as TNM stage which was classified using the 7th American Joint Committee on Cancer (AJCC) staging system for CRC and overall survival status [20]. None of the patients received radiotherapy, chemotherapy, or immunotherapy prior to enterectomy.

### One-step qPCR analysis

18 fresh CRC cancer tissue samples and 18 normal tumor-adjacent tissue samples were collected from the Department of Pathology, Affiliated Cancer Hospital of Nanjing Medical University for qPCR analysis. Total RNA extraction, quality control, and one-step qPCR analysis were performed as previously described [21, 22].

The primers for Rab27A were as follows: forward primer 5’- GTA AGT GAC ATA GTA GTT -3’ and reverse primer 5’- TTA TTC GTA GGT CTA ATG -3’. The glyceraldehyde 3-phosphate dehydrogenase (GAPDH) mRNA level was employed to standardize the measurements of the target gene and the primers for GAPDH were as follows: forward primer 5’-TGC ACC ACC AAC TGC TTA GC-3’ and reverse primer 3’-GGC ATG GAC TGT GGT CAT GAG-5’. Amplification conditions consisted of 30 min at 42 °C for reverse transcription and 2 min at 94 °C for Taq activation, followed by 35 cycles at 95 °C for 20 s, 56 °C for 20 s, and elongation at 72 °C for 30 s. Each measurement was performed in triplicate.

### Tissue microarrays (TMA) construction and IHC analysis

112 CRC and 113 normal tumor-adjacent tissues were enrolled in this present study. TMA was produced by the Department of Pathology, the Affiliated Hospital of Nantong University. Core tissue biopsies (2 mm in diameter) were taken from individual paraffin-embedded sections and arranged in the new recipient paraffin blocks. TMA was cut into 4-μm sections and placed on super frost charged glass microscope slides. IHC analysis was performed as previously described [23–25]. Deparaffinized sections (4 μm thick) from array blocks were separately stained on an Autostainer Universal Staining System (LabVision, Kalamazoo, MI, USA) using mouse anti-Rab27A antibody (1:100, Abcam, Cambridge, UK). The secondary antibody used was horseradish peroxidase-conjugated antibody (Dako Cytomation, Carpinteria, CA, USA).

Rab27A immunostaining observation and evaluation were simultaneously performed by two independent pathologists. IHC results were analyzed according to a previously described method [24, 26, 27]. Staining intensity was scored as follows: 0 (negative), 1 (weakly positive), 2 (moderately positive), and 3 (strongly positive). The percentage of Rab27A-positive cells was also classified into 4 categories, where 1 was given for 0-10 %, 2 for 11-50 %, 3 for 51-80 %, and 4 for 81-100 %. The product of the intensity and percentage scores gave rise to the final Rab27A staining score. The degree of Rab27A staining was sorted by a two-level grading system, and staining scores were described as follows: a score below 4 (<4) suggested low or no expression of Rab27A, while a score above 4 (≥4) suggested high expression of Rab27A.

### Statistical analysis

The Rab27A mRNA expression normalized to GAPDH in CRC samples compared with matching non-cancerous tissue samples was analyzed with the Wilcoxon non-parametric signed-rank test. The relationships between Rab27A expression and clinicopathologic itemss of CRC were evaluated with χ2 tests. Survival curves were performed with Kaplan-Meier method. Univariate and multivariate Cox regression model were performed to detect the prognostic elements. For all tests, the significance level for statistical analysis was set at *p* < 0.05. All data were analyzed using STATA Version 12.0 ((Stata Corporation, College Station, TX, USA).

## Results

### Summarization of clinical data of 112 CRC patients

A total of 112 CRC cases were collected from 73 men and 39 women and the representative clinical data are summarized in Table 1. The mean age of all cases at the time of surgery was 65.14 years. There were 47 cases with tumor diameter ≥5 cm, 62 with tumor diameter <5 cm. The tumor locations of 57 patients were in colon, 51 patients in rectum and 4 patients in ileocecal junction. The histological type of tumor in 102 cases was adenocarcinoma, while the other 10 cases were identified as non-adenocarcinoma type. 1 patient encountered a well-differentiated tumor, 92 suffered moderate tumor differentiation, and 15 had poor tumor differentiation. High serum CEA level (≥15 ng/ml) was observed in 14 cases, whereas low serum CEA level (<15 ng/ml) was displayed in 71 cases. The number of patients with T1, T2, T3 were 7, 26 and 79 respectively. Positive lymph node metastasis was observed in 44 patients while distant metastasis was noticed in 5 patients. According to TNM staging system, 29 patients were in stages I, 36 patients were in stages II, 42 patients were in stages III, whereas the remaining 5 patients were in stages IV.

**Table 1:** Correlation of Rab27A expression with clinicopathological characteristics of 112 CRC patients

Groups	No.	Rab27A		χ^2^	p value
		+	%		
Gender				1.1835	0.277
Male	73	39	53.4		
Female	39	25	64.1		
Age (years)				0.0133	0.908
≥60	74	42	56.8		
<60	38	22	57.9		
Tumor size (cm)				0.4585	0.498
≥5	47	25	53.2		
<5	62	37	59.7		
Insufficient data	3	2			
Tumor location				1.9870	0.370
Colon	57	32	56.1		
Rectum	51	31	60.8		
Ileocecal junction	4	1			
Histological type				2.3425	0.126
Adenocarcinoma	102	56	54.9		
Non-adenocarcinoma	10	8	80.0		
Tumor differentiation				3.3634	0.186
Well	1	0	0.0		
Moderately	92	55	59.8		
Poorly	15	6	40.0		
Insufficient data	4	3			
Serum CEA level (ng/ml)				0.2855	0.593
≥15	14	7	50.0		
<15	71	41	57.7		
Insufficient data	27	16			
T (Primary tumor)				3.0490	0.218
T1	7	6	85.7		
T2	26	16	61.5		
T3	79	42	53.2		
N (Lymph node metastasis)				5.2438	0.022*
Positive	44	31	70.5		
Negative	68	33	48.5		
M (Distant metastasis)				0.6280	0.428
Positive	5	2	40.0		
Negative	107	62	57.9		
TNM stage				9.2855	0.026*
Stage I	29	18	62.1		
Stage II	36	14	38.9		
Stage III	42	30	71.4		
Stage IV	5	2	40.0		

*p < 0.05

### Detection of Rab27A mRNA expression by qPCR test

**Fig. 1:**
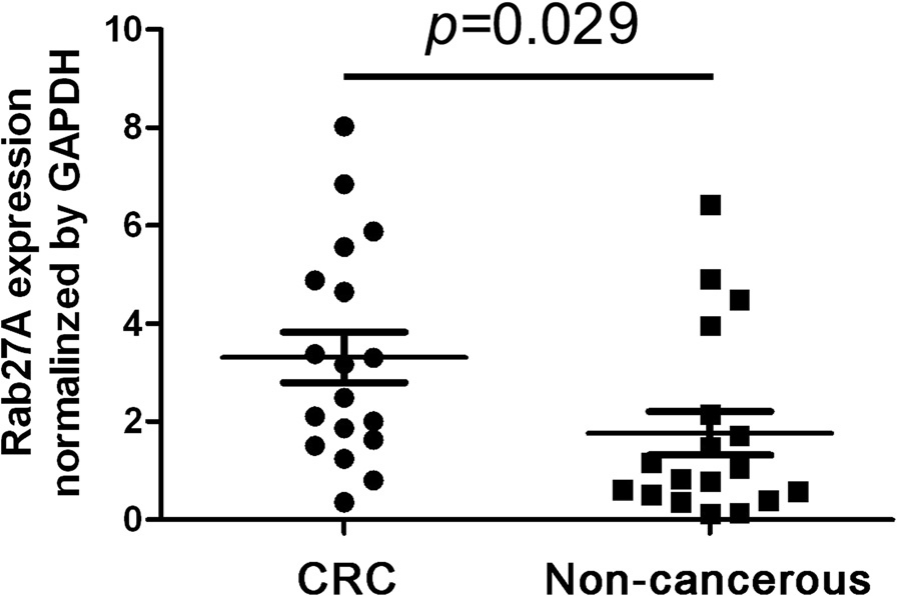
One-step quantitative real-time polymerase chain reaction (qPCR) was employed to evaluate Rab27A mRNA expression levels in 18 colorectal cancer (CRC) tissues compared with matched non-cancerous tissues. When normalized to glyceraldehyde 3-phosphate dehydrogenase (GAPDH) mRNA levels, the Rab27A mRNA level in CRC tissues (3.32 ± 0.518) is significantly higher than that in corresponding non-cancerous tissues (1.76 ± 0.444)

Total RNA was extracted from 18 CRC tissues as well as matched tumor adjacent tissues, and then subjected to one-step qPCR to detect Rab27A mRNA expression. When normalizing to GAPDH, the means of Rab27A mRNA in CRC tissues and that of the corresponding non-cancerous tissues were calculated as 3.32 ± 0.518 and 1.76 ± 0.444, respectively (t = 2.281, *p =* 0.029). Rab27A expression in the CRC samples was nearly 1.89-fold higher than that in matched non-cancerous tissues (Fig. 1).

### Detection of Rab27A protein expression by IHC

**Fig. 2:**
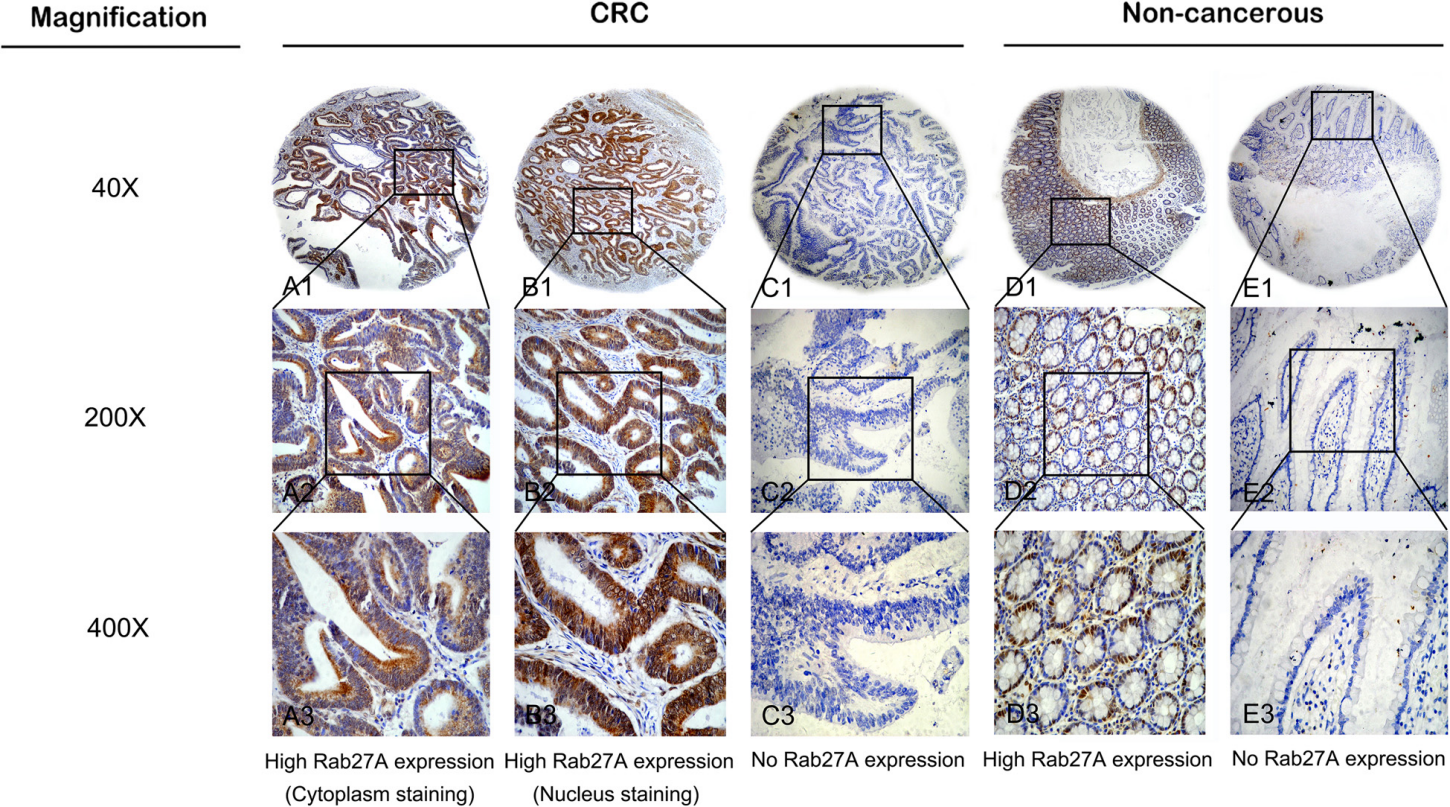
Representative images of Rab27A protein expression in colorectal cancer (CRC) tissues and corresponding non-cancerous tissues with tissue microarray (TMA) by immunohistochemistry (IHC) analysis. A1, A2 and A3 High IHC staining of Rab27A protein in cytoplasm of CRC cells. B1, B2 and B3 High IHC staining of Rab27A protein in nucleus of CRC cells. C1, C2 and C3 No IHC staining of Rab27A protein in CRC cells. D1, D2 and D3 High IHC staining of Rab27A protein in non-cancerous cells. E1, E2 and E3 No IHC staining of Rab27A protein in non-cancerous cells. Original magnification × 40 in A1, B1, C1, D1 and E1; ×200 in A2, B2, C2, D2 and E2; ×400 in A3, B3, C3, D3 and E3

High Rab27A expression was observed in 64 (57.1 %) of the 112 CRC samples compared with 47 (41.6 %) of 113 matched normal tumor-adjacent tissue samples. The difference was of statistically significance (χ^2^ = 5.44, *p* = 0.020) according to the χ^2^ test analysis and the data was agreeable with the previous qPCR test in which high level of Rab27A mRNA expression was detected. As is shown in Fig. 2, positive IHC staining was mainly localized in the cytoplasm and nucleus of CRC cells.

### Association between Rab27A protein expression and clinicopathological items

The association between high Rab27A protein expression and representative clinicopathological items was summarized in Table 1. High Rab27A protein expression was statistically related to N (*p* = 0.022) and TNM stage (*p* = 0.026). Relatively, other clinical items, such as gender, age, tumor size and location, histological type and tumor differentiation, serum CEA level and N, were rarely correlated with high Rab27A protein expression (Table 1).

### Survival analysis

**Fig. 3:**
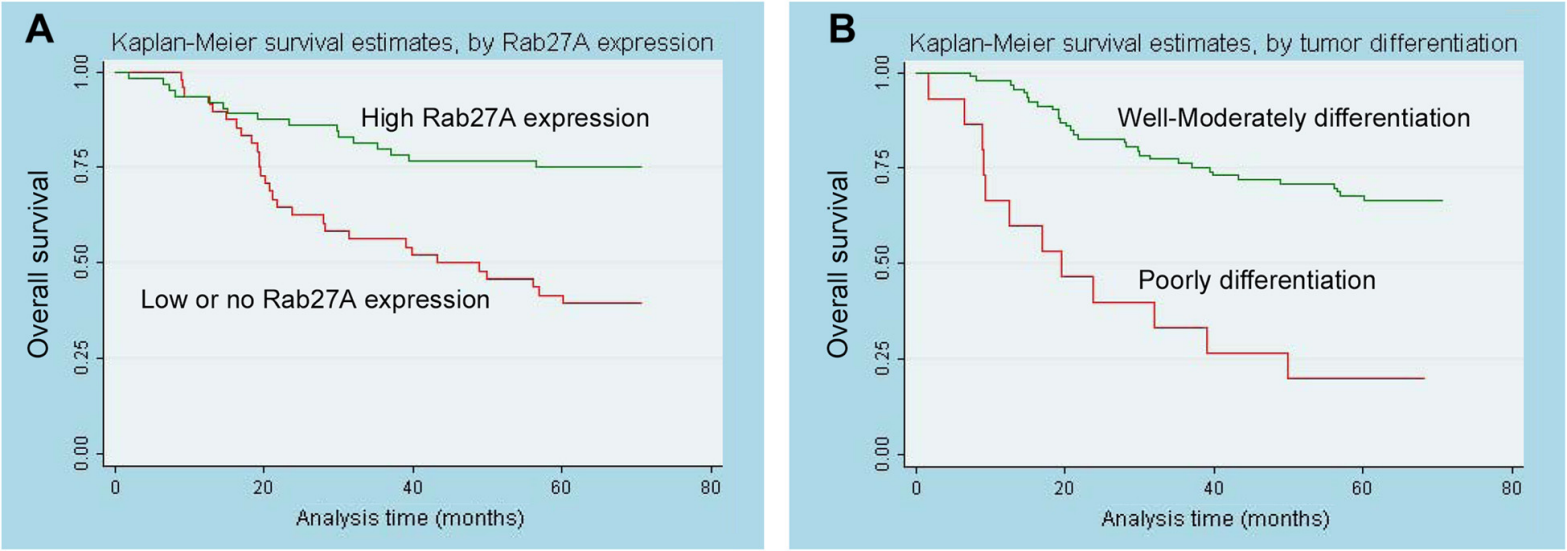
Survival analysis of 112 colorectal cancer (CRC) patients by Kaplan-Meier method. **a** Overall survival rate in patients with high Rab27A protein expression (green line) was significantly higher than that in patients with low or no Rab27A expression (red line). **b** Overall survival rate in patients with well-moderately differentiation of tumor (green line) was significantly higher than that in patients with poorly differentiation of tumor (red line)

According to univariate analysis, several factors were correlated with overall survival of 112 CRC patients, including Rab27A protein expression (*p* = 0.001), tumor differentiation (*p* = 0.001), serum CEA level (*p* = 0.006), T (*p* = 0.004), M (*p* = 0.005) and TNM stage (*p* = 0.003). Moreover, multivariate analysis was executed and the results showed that Rab27A protein expression (*p* = 0.012) and tumor differentiation (*p* = 0.004) were two independent predictors of overall survival (Table 2). Kaplan-Meier survival curves in Fig. 3 further demonstrated that CRC patients with high Rab27A protein expression and well-moderately tumor differentiation encountered significantly favorable survival time.

**Table 2:** Univariate and multivariate analysis of prognostic factors in 112 CRC patients for overall survival

	Univariate analysis			Multivariate analysis		
	HR	*p* value	95 % CI	HR	*p* value	95 % CI
Rab27A protein expression						
High versus Low or no	0.33	0.001*	0.181–0.616	0.36	0.012*	0.159–0.794
Gender						
Male versus Female	1.43	0.277	0.750–2.726			
Age (years)						
≥60 versus <60	1.16	0.642	0.618–2.184			
Tumor size (cm)						
≥5 versus <5	1.21	0.537	0.659–2.224			
Tumor location						
Colon versus Rectum versus Ileocecal junction	1.49	0.131	0.888–2.495			
Histological type						
Adenocarcinoma versus Non-adenocarcinoma	2.40	0.227	0.581–9.903			
Tumor differentiation						
Well-Moderately versus Poorly	0.23	0.001*	0.117–0.449	0.30	0.004*	0.134–0.689
Serum CEA level (ng/ml)						
≥15 versus <15	2.83	0.006*	1.356–5.894	1.64	0.228	0.735–3.639
T (Primary tumor)						
T1 versus T2 versus T3	0.31	0.004*	0.140–0.692	0.70	0.566	0.201–2.406
N (Lymph node metastasis)						
Positive versus Negative	1.41	0.257	0.780–2.533			
M (Distant metastasis)						
Positive versus Negative	4.35	0.005*	1.542–12.171	2.53	0.281	0.467–13.772
TNM stage						
Stage I versus Stage II versus Stage III versus Stage IV	0.58	0.003*	0.403–0.829	0.68	0.281	0.333–1.375

*p < 0.05

## Discussion

Recently, the roles of several members of the Rab family in human cancer have drawn great attention, including Rab23 and Rab25 [28–30]. As for Rab27A, high expression of Rab27A promoted invasiveness and metastasis of breast cancer cells by secretion of insulin-like growth factor-II [14]. Rab27A modulated the tumor microenvironment and facilitated tumor development by regulating exocytosis of multivesicular endosomes and lead to exosome secretion [17]. For glioma, Rab27A acted as an oncogenic factor and significantly associated with tumor progression and poor prognosis in all grades of gliomas [18]. Similarly, in HCC, Rab27A expression was closely correlated with HCC progression and can be used as a valuable prognostic indicator for HCC patients [19]. Hence it is rationale to presume that Rab27A also performs in CRC and there are certain relationships between Rab27A expression and certain clinical features of CRC patients.

The results of qPCR test with small samples of CRC showed that Rab27A expression in CRC samples was statistically increased than that in non-cancerous tissues. In the subsequent IHC analysis, the results were consistent with the previous qPCR test and the protein expression of Rab27A in CRC TMA was also higher than that in non-cancerous tissues. The data of differential expression of Rab27A in CRC in this present study was consistent with the content of previous reports which stated that expression of Rab27A was significant higher in glioma and HCC [18, 19]. In addition, high Rab27A protein expression do correlated with certain substantial clinical attributes, including N and TNM stage. The above data agreed with previous researches and supported the promotive function of Rab27A in tumorigenesis [16, 18, 19].

In the survival analyses of our present study, univariate analysis revealed that several substantial items correlated with overall survival of 112 CRC patients. Multivariate analysis further elucidated that Rab27A protein expression and tumor differentiation may identified as two independent prognostic factors for CRC prognosis in this present study. Thoroughly, Kaplan-Meier curve indicated that CRC patients with high Rab27A expression had a significantly better prognosis than that of patients with low or no expression. The data of survival analysis were in line with the previous study, which mentioned the prognostic effect of Rab27A in GC and CRC (without providing the exact survival data) [19].

So far, there are still some inconsistencies even contradictories concerning the effects of Rab27A in human cancers. Hendrix et al. reported no expression of Rab27A was detected in breast cancer [31] while Dong et al. reported that Rab27A expression was higher in primary HCC than in matched adjacent tissue [19]. High expression of Rab27A showed poor survival in gliomas and HCC as well as suggested favorable survival in GC and CRC simultaneously [18, 19]. In my view, these discrepant findings indicate that Rab27A may have different molecular mechanisms in different human cancer under specific circumstances. For instance, the secretion of exosome critically needs the present of Rab27A and the functions of tumor exosomes were dual. For one thing, exosomes could transfer tumor antigens to dendritic cells for presentation of these antigens to T lymphocytes [32]. For another, exosomes showed inhibitory functions on effector immune responses as well as promoted metastasis [33, 34]. The multiple activities of exosome may explain the complicated function of Rab27A in human cancer. Further researches that enroll larger cancer samples and elucidate the potential mechanisms of Rab27A performance are of great importance to prove our presumption.

In summary, to our best knowledge, this study was the first to report on the differential expression of Rab27A in CRC and our data indicated that Rab27A may be identified as a prognostic biomarker in CRC patients.

## Conclusions

In conclusion, this is the first report of the differential expression of Rab27A in CRC. High Rab27A expression was detected in CRC tissues, and that CRC patients with elevated Rab27A expression were prone to suffer malignant behaviors including positive N and advanced TNM stage. Moreover, high expression of Rab27A suggested favorable prognosis in CRC patients.
